# Rapid and precise diagnosis of disseminated *T.marneffei* infection assisted by high-throughput sequencing of multifarious specimens in a HIV-negative patient: a case report

**DOI:** 10.1186/s12879-018-3276-5

**Published:** 2018-08-07

**Authors:** Yi-Min Zhu, Jing-Wen Ai, Bin Xu, Peng Cui, Qi Cheng, Honglong Wu, Yi-Yi Qian, Hao-Cheng Zhang, Xian Zhou, Li Xing, Renhua Wu, Yongjun Li, Wen-Hong Zhang

**Affiliations:** 1 Department of infectious disease, Huashan Hospital of Fudan University, 12 Wulumuqi Zhong Road, Shanghai, 200040 China; 2 Binhai Genomics Institute, Tianjin Translational Genomics Center, BGI-Tianjin, BGI-Shenzhen, Tianjin, 300308 China; 3 BGI-Shenzhen, Shenzhen, China; 4 Wuhan National Laboratory for Optoelectronics, Huazhong University of Science and Technology, Wuhan, 430074 Hubei China

**Keywords:** *Talaromyces marneffei*, Fungi, Next generation sequencing, High throughput sequencing, Sterile body fluids

## Abstract

**Background:**

*Talaromyces marneffei*, is an opportunistic pathogenic fungus that is most commonly reported in Southeast Asia and disseminated *T.marneffei* infection predominantly occurs in patients with immunodeficiency. With a potential to invade multiple organs, it can be fatal for patients if diagnosis and treatment are delayed. In current clinical practice, the diagnosis of *T.marneffei* infection relies heavily on tissue culture and histologic analysis, which may suffer from limited positive rate and is sometimes time consuming. The rapid and accurate diagnosis of disseminated *T.marneffei* infection remains challenging.

**Case presentation:**

A 22-year-old man gradually developed fever, cough, lower extremities weakness, jaundice and rash, for which a 3-month extensive investigation failed to reach a diagnosis. After admitted into our hospital, laboratory and radiological tests revealed multiple lesions in the patient’s brain, spinal cord, and lungs. We performed next generation sequencing on the patient’s skin tissue, bone marrow, blood and cerebrospinal fluid, which all identified numerous *Talaromyces marneffei* nucleotide sequences and leaded to the rapid diagnosis and treatment of disseminated *T.marneffei* infection.

**Conclusions:**

This case underline the clinical significance of *T.marneffei* as a possible pathogen in immune-competent patients. This successful application of the next generation sequencing assisting the rapid diagnosis of disseminated *T.marneffei* infection provides a new perspective in the clinical approach to the systematic fungi infections and highlights the potential of this technique in rapid etiological diagnosis.

**Electronic supplementary material:**

The online version of this article (10.1186/s12879-018-3276-5) contains supplementary material, which is available to authorized users.

## Background

*Talaromyces marneffei*, formerly known as *Penicillium marneffei*, is a pathogenic, thermal dimorphic fungus that is most commonly reported in Southeast Asia. *T.marneffei* can cause disseminated infections and invade multiple organ systems such as blood, bone marrow, central nervous system, lungs and skins, therefore can be fatal for patients if not diagnosed and treated in time. Disseminated *T.marneffei* is known to predominantly occurr in patients with immunosuppression, such as HIV patients and patients receiving monoclonal antibody treatment [1–3], and is seldomly reported in non-HIV patients. The standard diagnostic method for *T.marneffei* infection mainly relies on tissue culture and histologic analysis, and previous studies have reported that whole blood nested polymerase chain reaction (PCR) and real-time PCR can provide an identification of the pathogen in HIV-infected patients [4]. Nevertheless, due to the low yield rate of tissue culture, the timely and accurate diagnosis of disseminated *T.marneffei* infection remains challenging.

## Case presentation

**Fig. 1:**
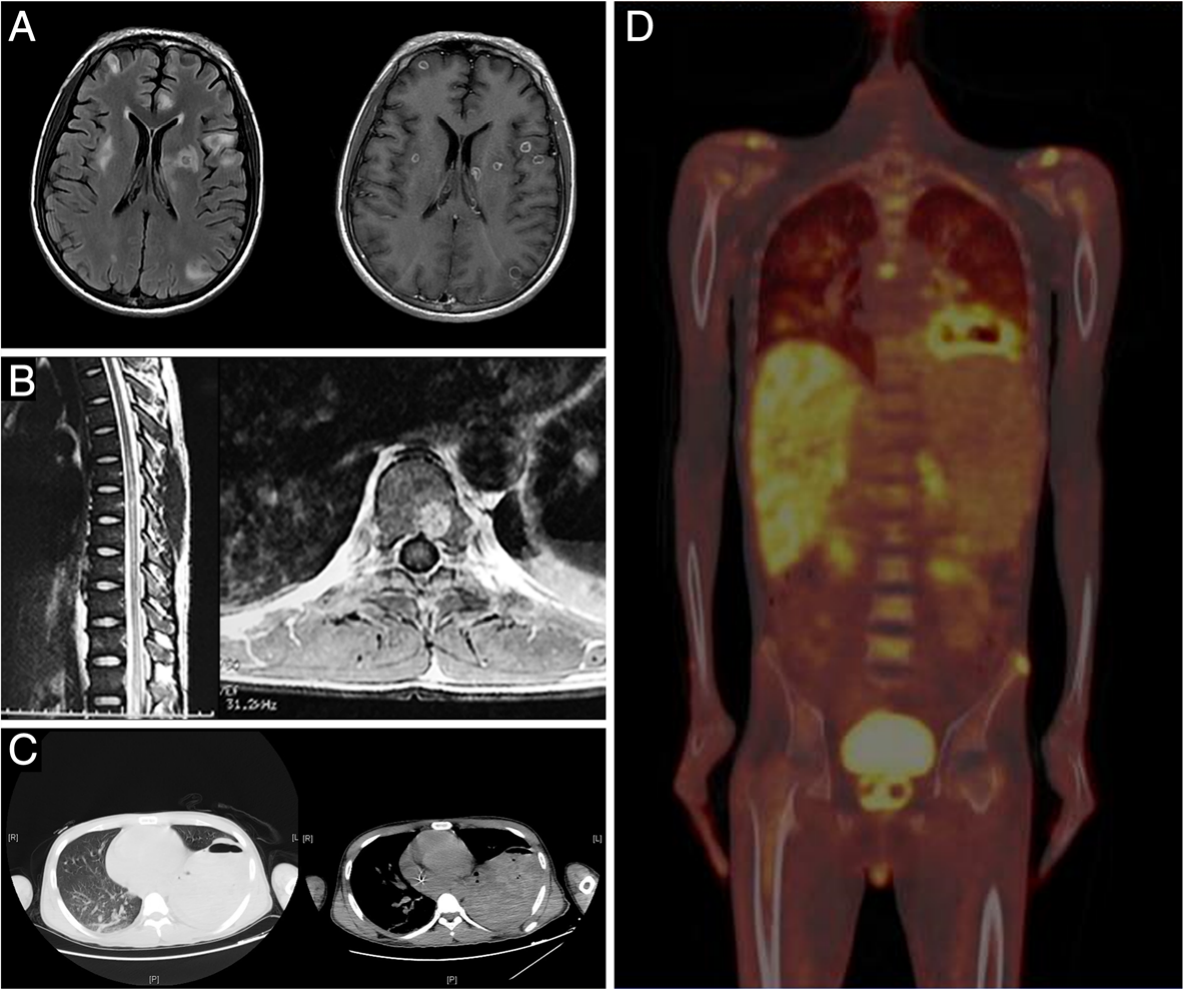
Imagine examination of the brain, spine, vertebra and lung: (**a**, **b**) Contrasted MRI were conducted on May 24th, 2017, and an axial T2-weighted image of the head found multiple lesions in the brain and abnormal signals were observed in spinal cord and vertebra in both sagittal and axial imaging. **c** Chest CT scan was conducted on May 28th and found multiple nodules in both lungs and a large lesion in lower left lung combined with cavity formation and gas-fluid levels inside. **d** PET/CT scan was conducted on June 10th and revealed abnormal uptake in the lesions of lung (SUV_max_ = 5.4), mediastinal lymph nodes (SUV_max_ = 4.4), liver (SUV_max_ = 3.8), left adrenal gland (SUV_max_ = 2.0), prostate (SUV_max_ = 6.6) and bones (SUV_max_ = 9.4). No abnormal uptake was found in brain

A 22-year-old man presented with a 70-day history of bilateral lower extremities weakness, followed by fever, productive cough and jaundice. On March 20th 2017, the patient suddenly developed lower limb weakness without any other notable symptoms. After the patient’s admission to the local hospital, brain computed tomography (CT) scan found no abnormalities while the contrast magnetic resonance imaging (MRI) detected multiple lesions in the brain and abnormal signals in spinal cord on T2 (Fig. 1a,b). Chest CT scan revealed multiple nodules in both lungs and a large lesion in lower left lung combined with cavity formation and gas-fluid levels inside (Fig. 1c). Abdomen CT scan showed hepatosplenomegaly and multiple retroperitoneal lymph node enlargement. Laboratory tests reported a leucocyte count of 12.7 × 10^3^ cells/μL, a alanine aminotransferase activity (ALT) of 116 U/L, a total bilirubin concentration (TBil) of 37 μmol/L (direct bilirubin (DBil): 31.8 μmol/L), an serum albumin level of 25 g/L and moderate anemia. HIV serology test, HBV/HCV serology test, serum T-SPOT.TB test, blood culture and cryptococcal latex agglutination test (CLAT) were all negative. Lumbar puncture revealed a decreased glucose concentration, an increased protein concentration and a normal leucocyte count of the cerebrospinal fluid (CSF). The results of bacterial and fungal cultures, CLAT, indian ink staining and acid-fast staining of CSF were all negative. The local hospital suspected disseminated tuberculosis infection and empirical anti-tuberculosis treatment with isoniazid, rifampin, ethambutol and pyrazinamide was prescribed.

After the treatment, no remission of the previous symptoms was observed and the patient developed new discomforts including fever (T_max_ 39 °C), productive cough and jaundice in the early April. Repeated blood, sputum and CSF cultures all came back negative. During the next two months, the patient continuously to have intermittent low grade fever, weakness of lower extremities and jaundice and by the end of May, rashes started to appear over his face and back. The local medical facility then transferred the patient to our hospital on June 7th, 2017.

**Fig. 2:**
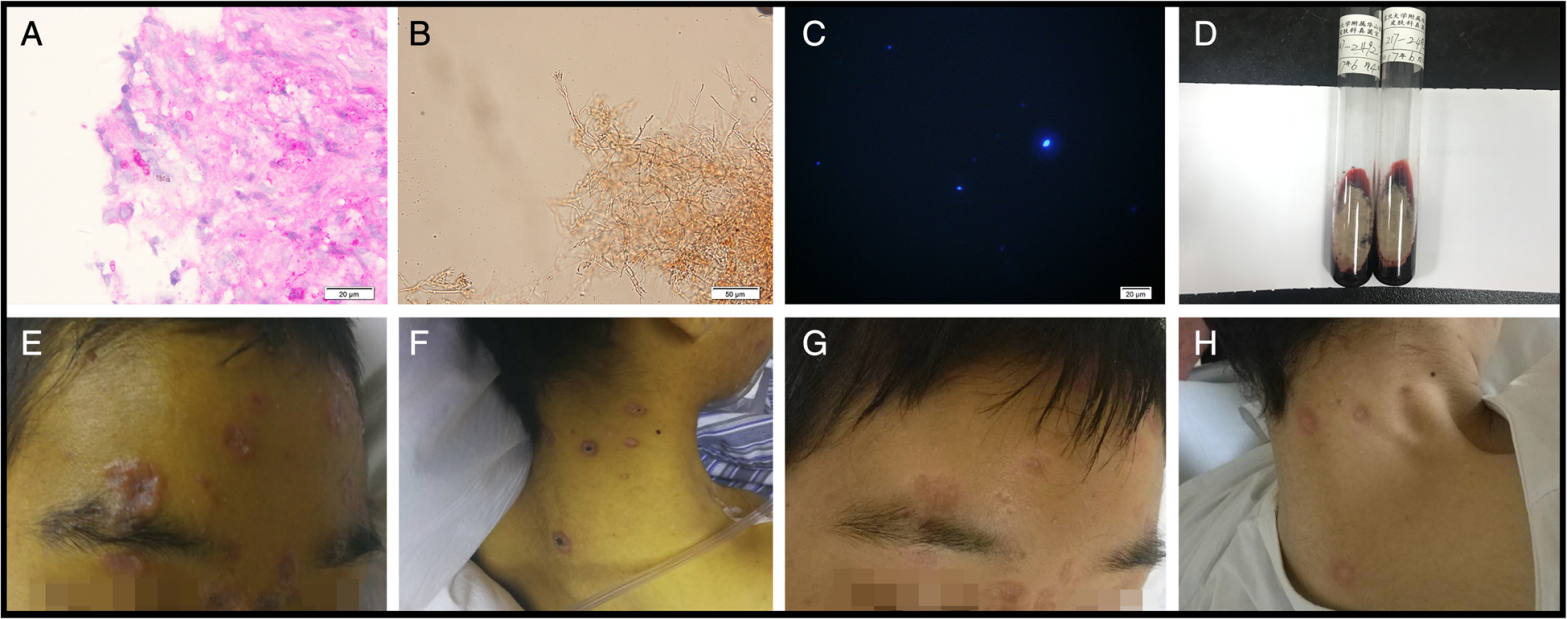
Skin biopsy, culture result and skin lesion: **a** Hematoxylin-eosin staining revealed spore-like structure in dermis. **b** KOH based smear revealed large amount of hypha in skin lesion. **c** Fluorescent staining confirmed the present of fungi in skin lesion. **d**. Skin tissue culture revealed that colonies at 25 °C produce a diffusible a wind-red pigment. **e**-**f**. Physical examination discovered multiple papules in the size between soybean and coin presented on the patient’s face and backside with umbilication and incrustation in the center of the lesion. **g**-**h**. The papules on the patient’s face and backside resolved after 3 weeks of treatment

A careful history was taken and the patient reported no significant past medical history and denied any history of addiction, drug abuse, and exposure to toxic matter. During examination, physician identified multiple papules on the patient’s face and backside, which were 0.5 cm to 2 cm in diameter with crusted center (Fig. 2). His skin and conjunctiva were yellow and both legs’ muscle strength were rated grade 0. He had slightly increased muscle tone and positive Babinski signs in both lower extremities. Laboratory testing revealed a C-reactive protein concentration of 29 mg/L, a ferritin concentration over 2000 ng/ml, an ALT of 36 U/L, an aspartate transaminase activity (AST) of 119 U/L, and a Tbil of 302.6. HIV RNA test were negative, and the lymphocyte subsets were within normal range. Blood gas analysis suggested a type II respiratory failure and other laboratory results were all insignificant. Contrast MRI scans revealed multiple lesions in the brain with ring enhancement, and abnormal enhanced lesions in spinal cord, pia mater spinals and vertebrae similar to previous imaging results. Contrasted CT scan further discovered suspicious lesions in liver, spleen, left adrenal gland, prostate, intra-abdominal lymph nodes and positron emission tomography/computed tomography scan found abnormal uptakes in these regions as well. After obtaining written consents from the patient, bone marrow biopsy and skin biopsy of the facial lesions were conducted on June 8th followed by lumbar puncture and fibro bronchoscope in later days. All samples were sent for culture and NGS (See Additional file 1 for NGS method) and during the following days, only supportive treatments were given.

**Fig. 3:**
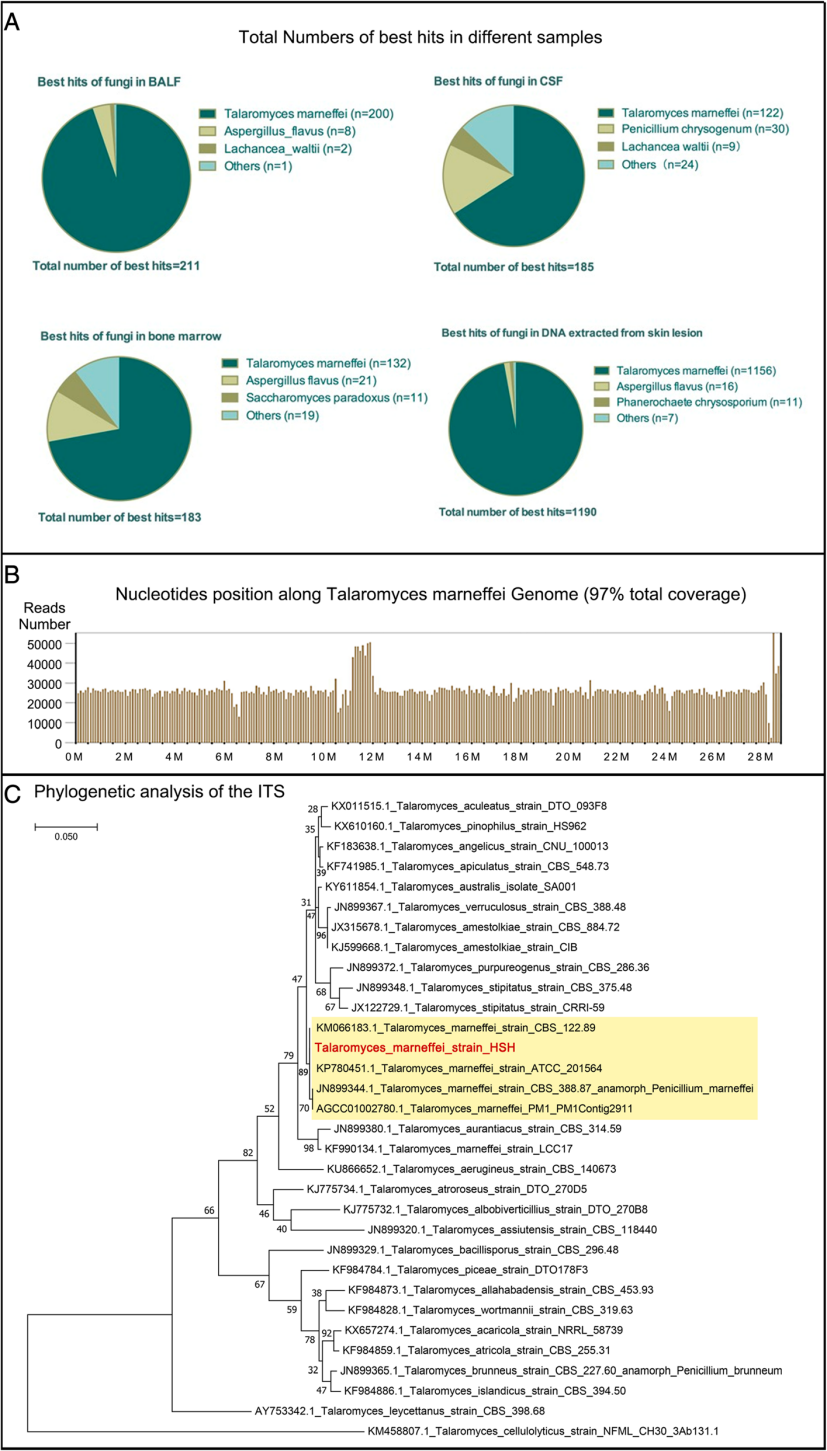
Sequencing results and Phylogenetic analysis: **a** The proportions of the identified sequencing reads among different clinical samples. The best hit is based on the highest alignment score between the query sequence and the database sequence segment. The *T.marneffei* owned the most best hits and was the most frequent detected species. **b** Sequencing of the isolated strain was conducted and a total coverage of 97% was obtained. **c** Phylogenetic analysis disclosed a close connection between the isolated strain and 4 other *T.marneffei* strains

On July 11th, NGS reported detecting 18,987 of 28,648,375 *T.marneffei* nucleotide sequences (Cover rate: 0.066%) in bone marrow and 144,780 of 28,648,375 *T.marneffei* nucleotide sequences (Cover rate: 0.51%) in DNA extracted from the skin lesions (Fig. 3a). Considering the notably high sequencing reads compared to the negative control (in which no *T.marneffei* nucleotide sequence was detected) and the patient’s clinical manifestation, physicians reached the diagnosis of *T.marneffei* infection and intravenous anti-fungi therapy consisting of 25 mg amphotericin B deoxycholate and 250 mg itraconazole per day was initiated. On July 14th, culture of the skin lesion tissue reported *T.marneffei* (Fig. 2) and 7 days later, repeated blood and bone marrow culture for bacteria, fungi and tuberculosis all came back negative. On July 19th, NGS reported detecting 25,906 of 28,648,375 *T.marneffei* nucleotide sequences (0.09%) in the BALF sample and 17,877 of 28,648,375 *T.marneffei* nucleotide sequences (0.062%) in the CSF sample. The cultures of both CSF and BALF for fungi and bacteria came back negative (Both Myco/F lytic system from BD company and Sabouraud’s agar medium were used in fungal culture).

We then conducted sequencing of the isolated strain from the skin lesion culture, and a 97% coverage of *T.marneffei* was identified (Fig. 3b). Close relationship between our strain and other *T.marneffei* strains was revealed in phylogenetic analysis (Fig. 3c). Confirmatory nested PCR [4] was done in patient’s blood, CSF, BALF and bone marrow samples and all PCR results were positive.

**Fig. 4:**
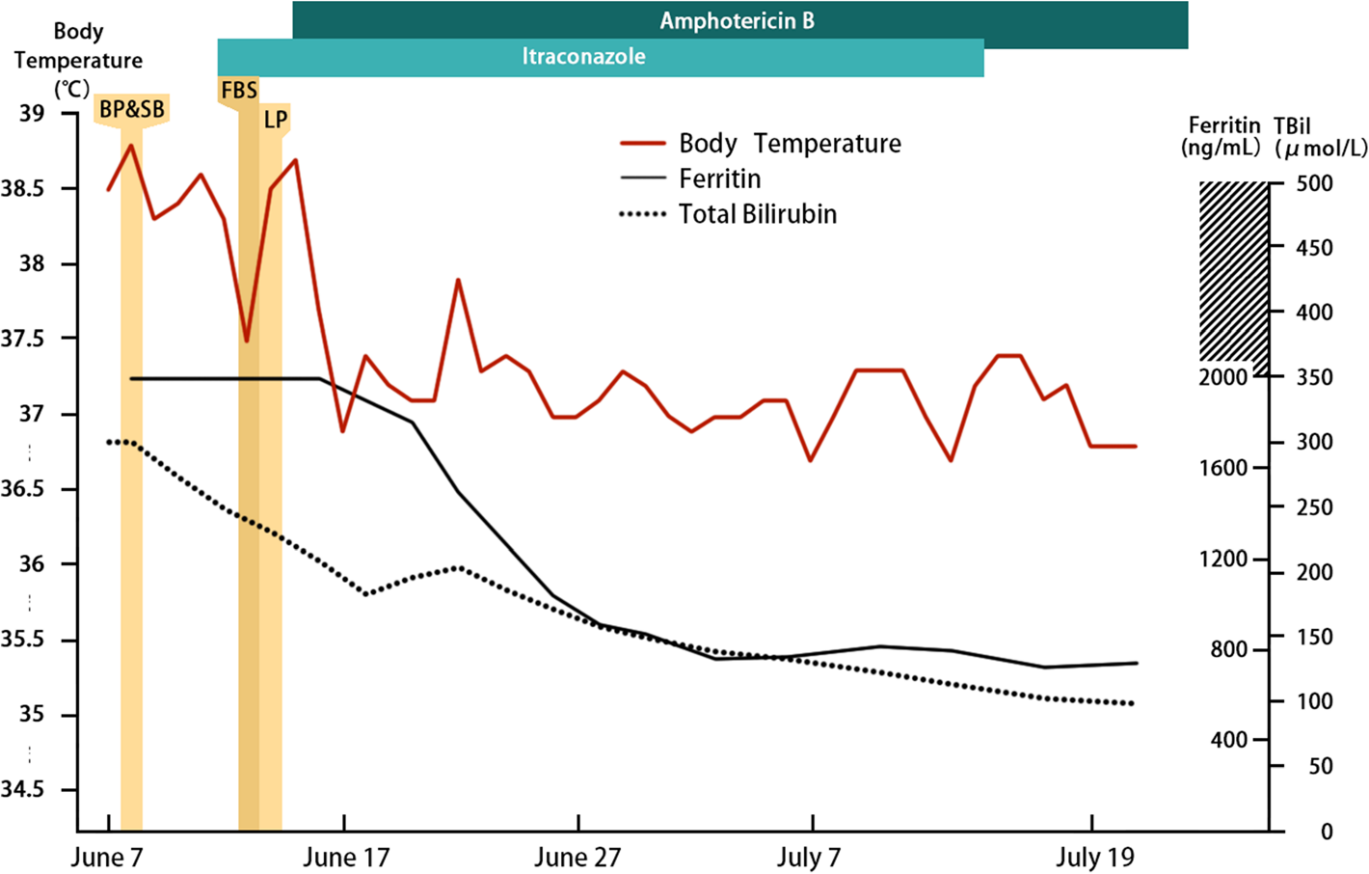
Clinical course the 22-year-old patient with disseminated *T.marneffei* infection: Fig. 4 shows the body-temperature curve line and the laboratory values of ferritin and total bilirubin obtained from the patient during the hospitalization. The vertical green lines shows the invasive procedures conducted, and the horizontal bars stands for the medications prescribed. BP bone marrow puncture, SB skin biopsy, FBS fibro bronchoscope, LP lumbar puncture

In consideration of all the facts, the physicians diagnosed the patient with disseminated *T.marneffei* infection, which invaded lung, bone marrow, central nervous system and skin. The anti-fungi therapy consisting of amphotericin B deoxycholate and itraconazole was continuously prescribed and the patient’s fever resolved three days after the initiation of the treatment, while other clinical symptoms such as cough, jaundice, skin lesions and lower limb weakness resolved within a month of the therapy (Fig. 4). Until this article was submitted, the total amount of amphotericin B deoxycholate reached 1170 mg, and the patient was discharged from the hospital with itraconazole oral solution.

## Discussion and conclusions

This is the first case report describing the diagnosis of disseminated *T.marneffei* infection in a HIV-negative patient with the assistance of unbiased NGS, which detected unique sequence reads corresponding to *T.marneffei* from multiple body fluids and tissues including bone marrow, CSF, BALF, and skin tissue. In this particular case, NGS identified *T.marneffei* as the causative agent without any prior assumptions from the physicians, leading to a prompt treatment and quick relieve of the disease. The sequencing data, which was in consistent with the patient’s clinical features and skin tissue culture result, finally assisted clinical physicians in approaching the diagnosis of disseminated *T.marneffei* infection.

Traditionally, the gold standard for infection diagnosis relies heavily on the isolation of pathogenic pathogen and therefore may suffer from drawbacks due to the limited culture positive rate [5]. Traditional PCR (including RT-PCR and nested PCR) or advanced multiplexed PCR assays provides advantages of high sensitivity, low cost and fast detection, however, the former method requires the physician to raise a few suspicious pathogens prior to examination and the latter method is usually restricted to only limited range of pathogens. Therefore, these methods are currently best applicable in certain clinical situations such as sepsis and respiratory infections [6, 7]. Under such circumstances, the implementation of NGS in clinical field enabled a fast and comparably accurate diagnostic tool for physicians, and most importantly, it does not require a predefined range of suspicious pathogens. The principles of NGS in infectious diseases are composed of three main procedures, the identification of the DNA nucleotides in the targeted samples, the comparison of these nucleotides against the catalogue of causative agents, and the decision-making progress whether the acquired sequences points to the possible etiological hypothesis [8]. The first study reporting using NGS in the diagnosis of infectious diseases was published in 2014, in a case of neuroleptospirosis in which 475 of 3,063,784 leptospira sequence reads (0.016%) were detected in the spinal fluid [9]. Since then, multiple studies have reported the use of NGS in central nervous system, bloodstream, and respiratory infections [10–13]. However, to our knowledge, few study has reported the use of NGS in the clinical fungi diagnosis.

One important limitation of NGS is the interference of the massive human nucleotide sequences during the data analysis. In a study of etiology diagnosis of NGS, the cell-free DNA (cfDNA) is extracted from plasma instead of whole blood for sequencing to maximize the percentage of sequences mapped to the pathogens. However, human reads still takes up around 95% of the total reads in the plasma samples [10]. In CSF, BALF and other body fluids, DNA rather than cfDNA is extracted from the sample and the studies have reported the detection of around 70–90% of the human reads [11, 12]. Hence, the filtration of the human nucleotide sequences during analysis is critical. Another major challenge of clinical use of NGS is how to interpret the results and determine whether the microorganism whose sequences are identified is truly the causative pathogen. The nature of NGS tends to detect all nucleotide sequences not only from the samples but also those acquired from the contamination during clinical procedures or laboratory processing, and thus NGS is limited in discriminating among colonization, infection or contamination. What’s more, identification of any nucleotide sequences of certain pathogens could not draw to the conclusion that this specific agents indeed exists in the sample, as the analysis merely indicate the mapping of a partial DNA fragment to a certain pathogen but could not retrieve its complete genome [8]. This may result in one read matching to multiple microorganisms due to the homology among them, as previous studies have shown [14]. Although some studies have reported the possible correlations between the number of pathogen-matched reads detected and the possibility of infection, few acknowledged explanation criteria of sequencing result has been established. Currently, there still lacks high-quality cohort study to reinforce this point and we now still rely on real-time PCR or other laboratory confirmation tests to assist NGS in the clinical approach.

This study provided a valuable case exploring the potential and possibility of NGS assisting the rapid clinical actionable diagnosis of *T.marneffei* and possibly other fungi from a multifarious body fluids and tissue types. Complementing the traditional laboratory and imaging tests, NGS may thus facilitate the precise diagnosis and the efficacious antimicrobial treatment in the field of clinical fungi infection.

## Additional file

Additional file 1: Methodology of the next generation sequencing and data analysis. The detailed methodology information of the next generation sequencing and data analysis adopted in this study. (DOCX 15 kb)

## Abbreviations

ALT: Alanine aminotransferase
AST: Aspartate transaminase
BALF: Bronchoalveolar lavage Fluid
cfDNA: Cell-free DNA
CLAT: Cryptococcal latex agglutination test
CSF: Cerebrospinal fluid
CT: Computed tomography
DBil: Direct bilirubin concentration
HBV: Hepatitis B virus
HCV: Hepatitis C virus
HIV: Human immunodeficiency virus
MRI: Magnetic resonance imaging
NGS: Next generation sequencing
PCR: Polymerase chain reaction
PET/CT: Positron emission tomography/computed tomography
SUV_max_: Maximum standardized uptake value
TBil: Total bilirubin concentration
