# Supplementary material for: Rapid and precise diagnosis of disseminated T.marneffei infection assisted by high-throughput sequencing of multifarious specimens in a HIV-negative patient: a case report
Source: BMC Infect Dis. 2018 Aug 7;18:379. doi: 10.1186/s12879-018-3276-5 (PMC6081951; doi:10.1186/s12879-018-3276-5)
Supplement: Supplementary file 1 — Methodology of the next generation sequencing and data analysis. The detailed methodology information of the next generation sequencing and data analysis adopted in this study. (DOCX 15 kb) [file 12879_2018_3276_MOESM1_ESM.docx]

**Next generation sequencing**

3mL of each sample (Bronchoalveolar Lavage Fluid (BALF), CSF and bone marrow) from the patient were collected according to standard procedures. 1.5mL microcentrifuge tube with 0.5mL sample and 1g 0.5mm glass bead were attached to a horizontal platform on a vortex mixer and agitated vigorously at 2800-3200 rpm for 30 min (For skin tissue, 10mg tissus plus 0.5ml H2O and 1g 0.5mm glass bead were attached to a horizontal platform on a vortex mixer and agitated vigorously at 2800-3200RPM for 30 min). 0.3mL sample was separated into a new 1.5mL microcentrifuge tube and DNA was extracted using the TIANAmp Micro DNA Kit according to the manufacturer’s recommendation. After synthesis of second-strand DNA, DNA libraries were constructed through DNA-fragmentation, end-repair, add A-tailing, adapter-ligation and PCR amplification. Agilent 2100 was used for quality control of the DNA libraries. Quality qualified libraries were sequenced by BGISEQ-100 platform.

**Data analysis**

High-quality sequencing data were generated by removing low-quality base calls from the 3' end of the read (Phred score <20), removing adapter, and discarding short (length < 35bp) reads. Then a filtering of human sequences was performed by mapping to the human reference genome (hg19) using Burrows-Wheeler Alignment. The remaining data were classified by simultaneously aligning to four Microbial Genome Databases in-house, consisting of viruses, bacteria, fungi, and parasites.

The classification reference databases were downloaded from NCBI (ftp://ftp.ncbi.nlm.nih.gov/genomes/). RefSeq contains 2,700 whole genome sequence of viral taxa, 1,494 bacteral genomes or scaffolds, 73 fungi related to human infection, and 47 parasites associated with human diseases.

The number of reads mapped to the microorganism was extracted while the coverage was calculated by dividing detected nucleotide sequences length by the length of whole genome (not calculated by reads number).We did not perform assembly, thus there is no need for validation. The reads number was standardized by the raw reads. Specific reads was defined as reads that doesn’t map to other microorganism with a same or a higher mapping score. The pathogens with at least 1 specific reads and a high cover rate compared with negative control was considered to be possible pathogen. And the final decision was made by considering the patient’s clinical manifestations.

**Results**

The specific reads number, specific reads percentage and the cover rate of *T.marneffei* detected in different samples was listed below. The relatively low reads percentage in Cerebrospinal Fluid was caused by the contamination of *Propionibacterium acnes*, whose reads number was 33139.

| Sample Type | Specific Reads Number | Specific Reads  Percentage (%) | Cover Rate |
| --- | --- | --- | --- |
| Bone Marrow | 126 | 56.25 | 0.066% |
| Cerebrospinal Fluid | 120 | 0.30 | 0.062% |
| Bronchoalveolar Lavage Fluid | 172 | 43.32 | 0.09% |
| Skin | 967 | 94.43 | 0.51% |
